# Sociocultural Factors Affecting Vocabulary Development in Young South African Children

**DOI:** 10.3389/fpsyg.2021.642315

**Published:** 2021-05-11

**Authors:** Frenette Southwood, Michelle J. White, Heather Brookes, Michelle Pascoe, Mikateko Ndhambi, Sefela Yalala, Olebeng Mahura, Martin Mössmer, Helena Oosthuizen, Nina Brink, Katie Alcock

**Affiliations:** ^1^Department of General Linguistics, Stellenbosch University, Stellenbosch, South Africa; ^2^Linguistics Section, School of African and Gender Studies, Anthropology and Linguistics, University of Cape Town, Cape Town, South Africa; ^3^Department of Health and Rehabilitation Sciences, University of Cape Town, Cape Town, South Africa; ^4^Department of Speech-Language Pathology and Audiology, Sefako Makgatho Health Sciences University, Pretoria, South Africa; ^5^Division of Speech-Language and Hearing Therapy, Department of Health and Rehabilitation Sciences, Stellenbosch University, Stellenbosch, South Africa; ^6^Department of Afrikaans and Dutch, North-West University, Potchefstroom, South Africa; ^7^Department of Psychology, Lancaster University, Lancaster, United Kingdom

**Keywords:** expressive vocabulary, CDI, sociocultural factors, South Africa, Afrikaans, isiXhosa, South African English, Xitsonga

## Abstract

Sociocultural influences on the development of child language skills have been widely studied, but the majority of the research findings were generated in Northern contexts. The current crosslinguistic, multisite study is the first of its kind in South Africa, considering the influence of a range of individual and sociocultural factors on expressive vocabulary size of young children. Caregivers of toddlers aged 16 to 32 months acquiring Afrikaans (*n* = 110), isiXhosa (*n* = 115), South African English (*n* = 105), or Xitsonga (*n* = 98) as home language completed a family background questionnaire and the MacArthur-Bates Communicative Development Inventory (CDI) about their children. Based on a revised version of Bronfenbrenner’s (1977) ecological systems theory, information was obtained from the family background questionnaire on individual factors (the child’s age and sex), microsystem-related factors (the number of other children and number of adults in the child’s household, maternal level of education, and SES), and exosystem-related factors (home language and geographic area, namely rural or urban). All sociocultural and individual factors combined explained 25% of the variance in expressive vocabulary size. Partial correlations between these sociocultural factors and the toddlers’ expressive vocabulary scores on 10 semantic domains yielded important insights into the impact of geographic area on the nature and size of children’s expressive vocabulary. Unlike in previous studies, maternal level of education and SES did not play a significant role in predicting children’s expressive vocabulary scores. These results indicate that there exists an interplay of sociocultural and individual influences on vocabulary development that requires a more complex ecological model of language development to understand the interaction between various sociocultural factors in diverse contexts.

## Introduction

Although there is a large and growing body of literature about sociocultural influences on child language development in many parts of the world, there is a dearth of knowledge on how different sociocultural factors interact and influence child language acquisition in African contexts. There are also no traceable studies comparing language acquisition of very young children across several linguistic and sociocultural contexts in southern Africa. Crosslinguistic studies on children’s vocabulary size typically focus on English-speaking children (mostly those growing up in the United States) and child speakers of one other language (Bornstein and Cote, 2005). In this study, we examine the influence of sociocultural factors on vocabulary development in toddlers aged 16 to 32 months across four different languages spoken in South Africa: isiXhosa^1^ and Xitsonga (Nguni and Thonga Bantu^2^ languages, respectively), and Afrikaans and South African English (SAE) (West-Germanic languages).

IsiXhosa is a Southern Bantu language grouped as S41 in Guthrie’s (1967/1971) classification. It is a Nguni language with a rich system of agglutinating morphology. Nouns belong to specific noun classes, indicated by a specific noun class prefix on the noun (Demuth et al., 2010), and by an agreement affix on the verb, the form of this affix being determined by the specific noun class prefix on the subject (Demuth et al., 2010). The verb complex is made up of a semantically meaningful stem, in combination with affixes that indicate grammatical characteristics and relationships such as subject and object agreement, tense-aspect, mood and negation, as well as various affixes such as the applicative and causative, which serve to introduce further arguments (see, e.g., Du Plessis and Visser, 1992; Zeller, 2008). Xitsonga is grouped as S53 in Guthrie’s (1967/1971) classification and is a cross-border language belonging to the Bantu-branch of the Niger-Congo languages. Concordial agreement between the preverbal subject and the verb is obligatory in most Bantu languages, but there is variation with respect to agreement with the object (Zerbian, 2007); in Xitsonga, such agreement with the object is not obligatory. As stated by Zerbian (2007), Xitsonga displays the structural properties common to Bantu languages, including a system of agglutinating morphology, and a rich noun class system overtly marked with noun class prefixes.

Afrikaans is a West Germanic language closely related to 16th century Dutch and is indigenous to South Africa. It is extremely impoverished on a morphological level in that there are no noun classes, noun prefixes or overtly marked subject-verb or object-verb agreement. Afrikaans does, however, mark plurals and past tense overtly by means of bound morphemes. Afrikaans shows word order variation due to, amongst others, scrambling and left dislocation (Biberauer, 2003). Along with Afrikaans, SAE is also a West Germanic language. Compared to isiXhosa and Xitsonga, SAE is highly impoverished on a morphological level, but not to the extent that Afrikaans is. For example, SAE displays subject-verb agreement, which Afrikaans does not.

These four languages were selected as they are typologically different, and the speaker base of each language is generally regarded as culturally different from the others. We make use of locally developed versions of the MacArthur-Bates Communicative Development Inventory (CDI)^3^, a comprehensive parental questionnaire which asks caregivers to indicate the words a child understands and produces across a wide variety of semantic domains (Fenson et al., 1993). We draw on an analytical framework that conceptualizes the impact of sociocultural factors on vocabulary development from an ecological systems theory perspective (see Bronfenbrenner, 1977), in which the individual’s development is understood in terms of interactions between different microsystems that can in turn be impacted by larger exo- or macrosystems. The individual has direct contact with the microsystem, such as their home environment. Components in the home microsystem, for example, household socioeconomic status, education of caregivers, and number of children and adults in the household shape everyday socialization practices. Interaction between the microsystems, such as home and school, make up the mesosystem that is in turn linked to and shaped by the larger exosystem. The exosystem relates to the individual without their involvement as an active participant, for instance the location/geographic area of the extended family and neighborhood, and larger economic and social influences. Everyday practices and activities making up different cultural environments both shape and are shaped by these different ecological systems that contribute in different ways to the individual’s developmental processes and outcomes (see Markus and Kitayama, 2009; Vélez-Agosto et al., 2017 on reinterpreting culture in Bronfenbrenner’s 1977 ecological systems approach).

### Known Influences on Vocabulary Development

Previous research points to several individual and environmental factors that influence a child’s general development. Similarly, vocabulary development can also be influenced by individual and environmental factors, including cultural aspects of the child’s environment (Tardif et al., 2008). Amongst the individual factors are age and sex: Vocabulary size increases with age (e.g., Maital et al., 2000 for Hebrew; Kern, 2007 for French; Bleses et al., 2008 for Danish; O’Toole and Fletcher, 2010 for Irish; Simonsen et al., 2014 for Norwegian). As regards sex, females fairly consistently demonstrate larger vocabularies than age-matched males (e.g., Fenson et al., 1994 for English). Stolarova et al. (2016) did not find differences between the vocabulary size of German-speaking males and females 2 years of age, but sex did influence vocabulary composition. For Spanish-speaking children of Mexican descent living in the United States, Jackson-Maldonado et al. (1993) found no sex differences for productive vocabulary. Yet Bornstein and Cote (2005) found uniform sex differences in expressive vocabulary size between 20-month-old speakers of three languages (Spanish, Italian, and American English) across three countries (Argentina, Italy, and the United States) and two geographic settings (urban vs. rural). Demonstrating an interplay between biological and environmental factors, Stolarova et al. (2016) found that 2-year-old females who did not attend a daycare regularly (i.e., who were cared for at home) had a slightly larger vocabulary than their male counterparts and also than boys and girls who did attend daycare regularly.

Other environmental factors that have been shown to influence language development in young children include socioeconomic status (SES) and maternal level of education, the latter at times used as a proxy for the former. Children from more affluent backgrounds have been found to demonstrate better language skills than those from poorer backgrounds (Hoff-Ginsberg, 1998; Reilly et al., 2010; Fernald et al., 2013). Studies have found more affluent children to have larger vocabularies (e.g., Hart and Risley, 1995), and to build their vocabularies at a faster rate (Hoff, 2003). SES does not, however, directly affect language outcomes^4^, such as vocabulary size; instead its effect is seen on children’s physical and psychological environments. These environments in turn affect children’s home learning environment (Attig and Weinert, 2020) and the language input they receive (see below), influencing the opportunities they have for vocabulary learning. SES can thus indirectly affect the child’s language experience – for instance, children who attend daycare centers with low teacher–child ratios (such as those found in more affluent areas) have been found to show more rapid development of grammatical skills (Burchinal et al., 2000). Also, adults and children in higher SES homes more frequently engage in joint book reading than those in lower SES homes (Coley, 2002; Attig and Weinert, 2020), and joint book reading has been shown to accelerate language development in a range of settings, both well-resourced and more poorly resourced (Whitehurst et al., 1988; Brown et al., 2018; Knauer et al., 2020), as has storytelling (Nicolopoulou et al., 2015).

The commonly used proxy for SES, maternal level of education, has been found to influence the language input children receive. Children of mothers with higher levels of education have been shown to demonstrate better language skills than their peers whose mothers have lower levels of education (Tomblin et al., 1991; Reilly et al., 2010; also see Hoff, 2003). In terms of productive vocabulary, several studies have shown that a higher level of maternal education correlates with a higher level of expressive vocabulary at different ages. McNally et al. (2019) found, using the British Ability Scales Naming Vocabulary Test with a nationally representative sample of children from the Republic of Ireland, that children of 36 months whose mothers had completed the minimum level of education had a mean vocabulary score almost 6 points lower than that of children whose mothers had a degree-level qualification. Although there is less work on the impact of maternal education on children’s expressive vocabulary in African settings, Vogt et al. (2015) reported comparable findings in a Mozambiquan sample using MacArthur-Bates CDIs: Children whose mothers had secondary education or higher produced significantly more words than children whose mothers only had primary education. Maternal level of education accounted for 2.6% of the variance in expressive vocabulary in the Vogt et al. study. However, maternal education did not account for differences in receptive vocabulary. Hoff-Ginsberg (1991) found differences in the language input that college-educated mothers and mothers who only completed high school provided to their children. The college-educated mothers used significantly more words, more different word types, and longer sentences than high school-educated mothers^5^. Hoff-Ginsberg (1991) also found that mothers who have a higher education level expanded their children’s utterances more during conversation with their children, make use of more partial self-repetitions and expansions, and ask more questions. Such linguistic behavior has been shown to benefit child language development (Hoff-Ginsberg, 1986), although there is a need for more research on caregiver–child interactions outside of WEIRD settings (where WEIRD refers to Western, Educated, Industrialized, Rich and Democratic; see Henrich et al., 2010). Findings in non-WEIRD contexts include those of Cristià et al. (2017), namely that in a community of forager−horticulturalists in Bolivia, children under 4 years of age are spoken to for less than 1 min per daylight hour. Similarly, Geiger and Alant (2005) found that in a village in Botswana, where they studied child-rearing practices and communicative interactions, there was little verbal interaction between mothers and children under the age of 5 years, and especially infants under the age of 1 year. The reason mothers provided for not conversing with the child during care activities (such as washing, dressing and feeding) was that the child could not yet speak. In fact, conversing with such young children was regarded as unusual and even unacceptable (also see Simonsen, 1990 for Western Samoa). Furthermore, most of the verbal interaction between caregivers in the village in Botswana and young children was instructional (often consisting of short behavior-directing commands from the adult’s side) with little verbal response required (or encouraged) from the child.

Taking note of the type of language a child hears in his/her microsystems is important because it could affect the child’s language skills. For instance, Huttenlocher et al. (2002) found that children who hear more complex language structures understand and produce more complex structures than those children who are exposed to simpler structures. Weisleder and Fernald (2013) found that the quantity of language infants hear correlates with vocabulary size at 24 months. The frequency with which words are heard also affects their acquisition order. For instance, Goodman et al. (2008) found that, within lexical categories, higher frequency of occurrence in parental, child-directed speech is related to earlier age of acquisition, when considering production data. One might predict that the quantity of language a child hears is directly related to the number of adults in the child’s environment who provide the child with language input, i.e., that more adults means more speakers and thus more language input (see Soderstrom et al., 2018). However, the context in which the adult–child interaction takes place can influence the quantity of language input the child receives. For instance, a sibling in the home may cause a single adult to direct less language to the child (see Oshima-Takane and Robbins, 2003), and, as stated by Soderstrom et al. (2018), an additional adult in the home could result in the child receiving less instead of more language input, as the adults may talk more to each other than to the child. Also, more people present could mean more talk not directed at the child, and more people talking simultaneously, thereby negatively affecting the amount of language that the child can process (Soderstrom et al., 2018). In contrast, Sperry et al.’s (2018) study of five American communities with different ethnic and SES backgrounds found a 17 to 58% increase in the number of words addressed to a child if one considers the input provided by all caregivers and not only by the primary caregiver, across the SES range studied. This suggests that having more, rather than fewer, interlocutors to provide language input may be beneficial for the child’s language development, in line with Weisleder and Fernald (2013).

Geographic location is a factor pertaining to the exosystem, outside of the individual’s microsystems. Where a child grows up geographically has been shown to influence the words to which the child is exposed and the number of words the child knows. Regarding types of words, the climate and terrain in the child’s environment might influence the terms a child knows for, *inter alia*, weather conditions, food and clothing variations, and types of fauna and flora. In terms of numbers of words varying across geographic locations, Bornstein and Cote (2005) found that Spanish-speaking urban children in Argentina and American English-speaking urban children in the United States have larger expressive vocabularies than their peers in rural areas. Bornstein and Cote (2005) discuss several ways in which rural life differs from urban life, and some of these differences might affect language learning. For instance, mothers in urban areas in Bali expect their children to acquire verbal assertiveness at a younger age than rural mothers do (Williams et al., 2000), which could influence socialization and other parenting practices, in turn influencing language exposure.

Remaining with geographic location as an exosystem-related factor shown to influence child language, Hamilton et al. (2000) found that British infants aged 1;0 to 2;1 have lower scores on both vocabulary comprehension and production than American infants of the same age assessed with a similar instrument (that of Fenson et al., 1994). Similarly, Bornstein and Cote (2005) found that Italian-speaking children in rural Italy had larger vocabularies than Spanish-speaking children in rural Argentina. After dismissing several possible reasons for their finding, Hamilton et al. (2000) speculate that the American infants’ higher vocabulary scores could be due to subtle cultural differences between the United Kingdom and the United States, such as differences between the two populations in terms of the number of children who attend daycare. In this regard, the duration of 2-year-olds’ daycare experience has been shown to correlate positively with their vocabulary size (Stolarova et al., 2016). Hamilton et al. (2000) also speculate that there could be differences between the British and American parents in terms of the frequency with which they use the words on the assessment instruments during child-directed speech, and that would influence the rate at which the children learn these words, as discussed above.

### Sociocultural Factors in South Africa

South Africa is a plurilingual country, with 11 official languages, each with subvarieties, as well as several other languages spoken across the country’s surface area of 1.22 million km^2^, with each language having more than one area of speaker concentration. Different regions of the country are associated not only with different language combinations but also with different sociocultural environments, even among speakers of the same language.

Microsystem-related factors in South African contexts that warrant special mention include SES and maternal level of education. There is a comparatively low level of education in South Africa: 6% of adults aged 25 to 64 years have had no schooling, 14% have at least some primary school education but no high school, and 68% went to high school but did not necessarily complete all high school grades (Statistics South Africa, 2017). Also, there is vast inequality in family income distribution in South Africa,^6^ and about half of South African adults live below the upper-bound poverty line (see Statistics South Africa, 2019). Many South African children are thus raised in low SES households, putting them at potential risk for poor language development. Further, South African children grow up in a variety of household structures, including nuclear family households (a couple with their own children only; 19% of the country’s households); single-parent households (a single parent with his/her own children only (11%), and extended households (36%) (Statistics South Africa, 2018). This translates to 25% of South African children living in nuclear households, whereas 62% live in extended households (see Hall and Mokomane, 2018). Most children co-reside with at least one of their biological parents, although large rural-urban differences exist: For instance, 21% of rural and 45% of urban children reside with both their parents whereas 30% of rural and 15% of urban children reside with neither of their parents. Where both parents are absent, the caregiving responsibilities are typically taken up by the grandparent(s) (68%), an aunt or another relative (19%), or siblings (7%) (see Hall and Mokomane, 2018). Whereas the number of adults could increase the quantity of language produced in the home, there is evidence that only child-directed speech (and not speech the child may overhear between adults) correlates with vocabulary size (see Weisleder and Fernald, 2013). Regarding the number of other children in the household, according to Havron et al. (2019), siblings may either compete for parents’ attention, thereby reducing the quantity of child-directed input any one child receives, or may, at least in part, make up for the lost input by themselves serving as a source of language input. In this regard, in the Bolivian community that they studied, Cristià et al. (2017) found that adults provide the majority of language input that children up to the age of 3 years receive, after which the proportion of input received from other children increases.

### Research Questions

To establish which sociocultural factors impact the expressive vocabulary of young South African children, we ask the following question: Do individual factors (age and sex), microsystem-related sociocultural factors (SES, maternal level of education, and number of other children and adults in the household), and exosystem-related sociocultural factors (home language spoken and geographic location, i.e., rural vs. urban) affect the size and composition of the expressive vocabulary of South African toddlers who speak Afrikaans, isiXhosa, SAE, or Xitsonga? We divide this question into three parts:

RQ1: What are the effects of the above-mentioned sociocultural factors on total vocabulary size at the individual, microsystem and exosystem level?

We hypothesize that being older, being female, and having a larger number of adults and children in the household, a mother with a higher level of education, and higher SES correlate with a larger expressive vocabulary.

RQ2: When all these sociocultural factors are considered together, how much of the variance in total vocabulary size can be accounted for?

We hypothesize that age, sex, number of adults and children in the household, maternal education, SES, and geographic location will all account for variance in total vocabulary size. Age is expected to contribute the most, and number of children the least.

RQ3: Do these sociocultural factors correlate with the vocabulary size in different semantic domains deemed to be common across the four languages concerned?

We hypothesize that geographic location will be correlated with most semantic domains as these domains might be susceptible to characteristics of an area, e.g., the type of animals encountered, or the types of foods eaten.

## Materials and Methods

### Research Design

This study has a quantitative design and is cross-sectional, crosslinguistic and descriptive in nature. Data for this paper were collected as part of a multilingual, multidisciplinary, inter-institutional research project on the gesture and language development of young South African children in all South Africa’s official languages (see Brookes et al., forthcoming; Dowling and Whitelaw, 2018). To obtain information on children’s language development for this paper, adapted MacArthur-Bates CDIs and a family background questionnaire for four of South Africa’s official languages were completed by the caregivers of Afrikaans-, isiXhosa-, SAE-, or Xitsonga-speaking toddlers of 16 to 32 months.

### Participants

Caregivers of 428 children aged 16 to 32 months were recruited via (i) local childcare institutions and local and national not-for-profit organizations offering services directed at families with young children, (ii) existing personal and professional networks of the researchers, and (iii) social media. Caregivers were either one of the child’s birth or adoptive parents, grandparents, other family members, or another guardian who parented the child alongside or instead of the biological parent. Inclusion criteria were that (i) the caregiver had to be a South African national (ii) raising a child of 16 to 32 months (iii) in their mother tongue (iv) in South Africa. The exclusion criteria were more than 4 h per day of exposure to another language/other languages in the child’s home, and caregiver concern about the child’s hearing or communication development. We excluded children who received more than 4 h a day of exposure to other languages to control for the often reported – and contested (see, e.g., Pearson et al., 1993; Hoff et al., 2012; De Houwer et al., 2014) – difference in expressive vocabulary size between monolingual and multilingual children when considering the vocabulary size in each of the multilingual child’s languages separately. We also wanted to avoid adding the variable of amount of exposure to each language, given that bilingual children have been shown to have higher vocabulary scores for what is reported to be their first than for their second language (O’Toole et al., 2017). Children for whom concerns about hearing and/or communication development were reported were excluded to limit the number of factors which could cause variation in vocabulary size in our sample, given that our focus was on sociocultural (and not health-related) influences.

Our sampling plan stated that half of the targeted 100 participants for each language had to be male, to control for the often-reported influence of sex on child language skills. For Afrikaans, isiXhosa and Xitsonga, half of the participants had to live in rural areas, to control for the reported effect of geographic location on vocabulary size and composition. For these three languages, there were no specific targets as regards SES. For SAE, half of the participants had to be from low SES homes, regardless of geographic location, because SAE is infrequently spoken as home language in rural areas, but does vary according to SES (see Mesthrie, 2002; Bekker, 2012). Table 1 shows the number of the participants and their demographic information. As can be seen from this table, the target number of participants was exceeded for all languages apart from Xitsonga. The Afrikaans participants had the highest mean age (1.32 months higher than the youngest language group, isiXhosa). Whereas SAE and isiXhosa each had almost the same number of male and female participants, Afrikaans had more females than males and Xitsonga more males than females. However, an ANOVA yielded no statistically significant group differences for Sex [*F*(3,424) = 1.104, *p* = 0.347] nor for Age [*F*(3,424) = 1.410, *p* = 0.239]. Afrikaans, Xitsonga, and isiXhosa collectively had 163 rural and 160 urban participants, and all but three SAE participants were situated in urban areas.

**TABLE 1:** Participant demographic information by language.

	Afrikaans	isiXhosa	SAE	Xitsonga	Total
Number of participants	110	115	105	98	428
**Child age (in months)**					
Range	16–32	16–32	16–32	16–32	16–32
Mean	24.33	23.01	23.66	23.44	23.61
SD	4.75	5.02	4.66	5.05	4.88
**Child sex**					
Female	61	57	53	42	213
Male	49	58	52	56	215
**Geographic setting**					
Rural	49	61	3	53	166
Urban	61	54	102	45	262

### Data Collection Instruments

The MacArthur-Bates CDI has been adapted into nearly 100 languages from a range of language families^7^. It has an infant version (on the gestures, play routines, common action, and words that children of 8 to 18 months can understand and use) and a toddler version (on the words and early morphology, word combinations and sentence complexity of children aged 16 to typically 30/36 months). For the purposes of this paper, only the word section of the toddler version was considered. In each case, the caregivers were asked to indicate on a checklist whether the child understood and produced the word. The South African versions of the CDI have not yet been validated. The question that can arise is whether caregivers in South Africa are able to report accurately on their toddlers’ language skills – if the caregivers engage in less child-directed speech, do they know their child well enough linguistically to reliably indicate which words their child understands and produces? Although South African data are not yet available, Alcock et al. (2015) found that in rural Kenya, caregivers were able to accurately report their younger children’s receptive vocabulary (at an age when there are few productive words to report) and older children’s grammatical errors. Based on this study from Kenya, we worked on the premise that South African caregivers are capable of providing reliable information.

The American English toddler version of the CDI (Fenson et al., 1993) was translated by three adult mother-tongue speakers per language. Hereafter, adaptations (entailing the addition or removal of words) were made based on the outcome of (i) a minimum of two focus group discussions and/or sets of interviews^8^ with parents of young children and professional child service providers, (ii) consultation with linguists and speech-language therapists who are mother tongue speakers of the language (five for Xitsonga, three for isiXhosa, three for Afrikaans, and two for SAE), and (iii) 30-min samples of naturally occurring speech from six children per language (see Brookes et al., forthcoming). The preliminary versions of the CDIs and family background questionnaires were piloted with 40 caregivers of 16- to 32-month-olds per language (for Afrikaans, Xitsonga, and isiXhosa, 20 rural and 20 urban; for SAE, 20 low- and 20 mid-SES). After this pilot, statistical analyses of the data obtained guided decisions on further exclusion or replacement of lexical items. From the approximately 1200 lexical items piloted, 733 to 773 vocabulary items per language were retained for the CDIs used in the current study. The CDIs of the West Germanic languages had one more semantic domain than the Bantu language CDIs, as pronouns were not included in the Bantu language CDIs^9^. For the current study, the total CDI vocabulary score and a subset of 10 semantic domains (amounting to approximately half of the total number of lexical items on the CDI) were used for analysis. This selection was made to reduce the number of semantic domains to a manageable number, as the scope of this article did not allow consideration of all semantic domains. These 10 domains were selected based on their similarity in terms of number of items across languages and their tangibility, in that they either are all nouns or refer to games and routines, which we expect would make them more susceptible to sociocultural differences (see, e.g., Potgieter and Southwood, 2016 for a South African study which found that 4-year-old low-SES and mid-SES monolingual children differed significantly in terms of their noun-related but not verb-related vocabulary scores). These 10 domains were ANIMALS, CLOTHING, FOOD AND DRINK, FURNITURE, GAMES AND ROUTINES, PEOPLE, PLACES TO GO, SMALL HOUSEHOLD ITEMS, TOYS, and VEHICLES. Table 2 contains selected information on the number of lexical items per language version of the CDI used for data collection for this paper.

**TABLE 2:** Number of lexical items of the CDI, by language and semantic domain.

	Afrikaans	isiXhosa	SAE	Xitsonga
Total number of semantic domains	22	21	22	21
Total number of lexical items	770	748	773	733
**Ten selected semantic domains**				
Animals	51	51	52	52
Clothing	33	34	33	34
Food and drink	74	73	74	72
Furniture	33	33	33	32
Games and routines	36	36	36	36
People	22	25	28	25
Places to go	18	18	18	18
Small household items	74	72	74	71
Toys	18	18	18	18
Vehicles	12	12	12	12
10 selected domains combined	371	372	378	370

The family background questionnaire was developed after consulting (i) the literature on demographic and other factors influencing language development in young children, (ii) the results of the 2011 South African census (Statistics South Africa, 2012), and (iii) members of communities speaking the language concerned. The questionnaire included questions on child health and development; childcare arrangements; household composition, income and food expenditure; parental level of education and occupation; and language exposure in and outside of the home, as these factors have been shown to affect child language development in other research contexts. Each language version of the questionnaire was piloted along with the CDI for that language, and questions were subsequently omitted, refined and rephrased based on the feedback received from the parents, caregivers and fieldworkers about their clarity, ease of reading, and cultural appropriateness.

### Data Collection Procedures

An electronic version of the consent form, family background questionnaire and CDI for each language was created on Qualtrics (Qualtrics, Provo, UT, United States), combined into one online form. The majority of the data were collected by fieldworkers who were either students or employees of child development organizations. They were trained online using Zoom or WhatsApp, as South Africa was in full to moderate lockdown due to COVID-19 at the time of data collection, and contact research was therefore not allowed. All data were collected either using the fieldworkers’ smartphones or tablets (using a link sent to them via WhatsApp), or – in cases where fieldworkers did not have their own suitable devices – on tablets couriered to them with the correct language version of the form in Qualtrics preloaded onto the tablet. Where assisted by a fieldworker, caregivers completed the questionnaire and CDI on their smartphones, with the fieldworker being available for consultation throughout. Caregivers without smartphones and/or sufficient literacy skills were interviewed telephonically by the fieldworker who entered the caregivers’ responses into Qualtrics. Cellphone credit and internet data to do so were supplied electronically to fieldworkers and caregivers. For some of the Afrikaans and SAE submissions, the electronic form was completed independently by the caregiver. In these cases, the caregivers had sufficiently high levels of literacy, and had access to a suitable electronic device and internet connection.

The consent form, questionnaire and CDI collectively took 40 to 60 min to complete, depending on the number of lexical items the child knew and the caregiver’s reading ability and computer literacy. Qualtrics allows completion across multiple sessions (and automatically takes one to the first uncompleted page if reopened on the same device), so caregivers were able to stop and resume as needed. Submission had to take place within a week of first opening the form on Qualtrics; opened but unsubmitted forms were submitted automatically by Qualtrics after a week.

### Ethical Considerations

Ethical clearance for the study was obtained from the relevant research ethics committees at the University of Cape Town and Stellenbosch University^10^. Information on the study and informed consent forms were available in the mother tongue of the participants on Qualtrics, and if consent for participation was not granted, Qualtrics did not allow the potential participant to proceed to the family background questionnaire and CDI.

The informed consent form, family background questionnaire and CDI were completed voluntarily and anonymously. Participants could withdraw from the study at any stage by exiting Qualtrics prematurely. Qualtrics records all responses and indicates the percentage completion of each form. Submissions not showing a 100% completion were removed during data cleaning, thereby effectively making it possible for participants to withdraw their data from the study.

Participants who completed the form independently donated their time to the research project. Those who completed the form with the assistance of a fieldworker could supply a mobile phone number to the fieldworker (not via Qualtrics) in order to be sent an electronic supermarket voucher as a thank-you gift (to the value of approximately 10 loaves of bread) via WhatsApp or text message. The research team ensured that all COVID-19-related social distancing protocols of their respective institutions were followed to protect both fieldworkers and participants from undue risk.

### Analytical Strategy

In order to address RQ1 and RQ2, hierarchical linear regression was conducted in R version 4.0.2 (R Core Team, 2020), using the *lm* function, to determine whether the selected sociocultural variables can predict the participants’ Total vocabulary score. Four separate blocks were applied, controlling for the variables entered into the previous blocks. Age was entered as the first control variable whereas the second block contained the other individual factor, Sex. The third block contained the microsystem factors (SES, Maternal education, Number of adults in the household, and Number of other children in the household), which refer to systems with which the child is said to have direct interaction, whereas the fourth block contained the exosystem factors (Geographic area, which referred to rural vs. urban area, and Language).

RQ3 was answered by calculating correlations, first for all languages combined and then for each language separately. This was done to determine whether any relationships exist between the above-mentioned sociocultural factors and the 10 semantic domains.

## Results

The mean expressive vocabulary score and descriptive statistics for the sociocultural factors for each language are shown in Table 3. Due to the number of items in the semantic domains being slightly different across languages, all scores were converted into percentages to ensure comparability. The mean vocabulary score for isiXhosa-speaking children (37%) was lower than for the other three languages at 46% for Afrikaans and SAE, and 48% for Xitsonga, and these group differences between vocabulary scores were significant [*F*(3,424) = 3.850, *p* = 0.010]. Age differences between the groups could not account for differences in expressive vocabulary size, because although the isiXhosa group’s mean age was lower than those of the other language groups (Table 1), the intergroup age difference was not statistically significant. Further investigation into possible reasons for the differences in vocabulary size falls beyond the scope of the current study.

**TABLE 3:** Descriptive statistics.

Language	Sociocultural factors	*N*	Range	Mean	SD
Afrikaans	Total vocabulary (%)	110	0–98.05	45.91	30.06
	Children in household	110	0–6	0.95	1.21
	Adults in household	110	1–7	2.84	1.22
	Maternal education	109	2–6	4.92	1.01
	SES composite score	110	1.92–9.09	6.27	1.82
SAE	Total vocabulary (%)	105	0–97.80	45.97	29.10
	Children in household	105	0–8	0.70	1.10
	Adults in household	105	1–8	2.74	1.32
	Maternal education	105	1–6	5.36	0.89
	SES composite score	105	2.86–9.39	7.25	1.44
isiXhosa	Total vocabulary (%)	115	0–84.76	36.76	23.77
	Children in household	115	0–6	1.47	1.51
	Adults in household	115	1–10	2.83	1.53
	Maternal education	112	2–6	4.55	0.93
	SES composite score	115	0–10	4.66	1.70
Xitsonga	Total vocabulary (%)	98	3–100	48.43	26.89
	Children in household	98	0–6	1.93	1.45
	Adults in household	98	1–8	3.19	1.39
	Maternal education	96	3– 6	4.96	0.81
	SES composite score	98	2.12–8.57	4.85	1.53

*Maternal Education scale: 1 = no formal schooling, 2 = primary school incomplete, 3 = completed primary school, 4 = high school incomplete, 5 = completed high school, 6 = post-school qualification. SES composite score based on maternal and paternal level of education, maternal and paternal employment status, household income, and household expenditure on food.*

Looking at household factors, the number of other children in the household ranged from 0 to 8, but the means ranged from 0.7 for SAE to 1.9 for Xitsonga, indicating that the children in our sample are growing up on average as one of two or three co-residing children. Regarding number of adults in the household, the range for all languages collectively was 1 to 10, with the mean for the Xitsonga group (3.2) being higher than those of the Afrikaans and isiXhosa groups (2.8), and the SAE group (2.7).

Maternal level of education was on a six-point scale, ranging from 1 (no formal schooling) to 6 (at least one completed post-school qualification). For all language groups, there were some mothers with post-school qualifications, but the lowest level of education differed: In the Xitsonga group, the mothers with the lowest level of education had completed primary school, whereas for Afrikaans and isiXhosa, there were some mothers who attended primary school without completing it, and there were mothers in the SAE group with no formal schooling.

Given the extent to which maternal level of education has been reported to correlate with child language skills, we first considered it as a separate sociocultural factor. Maternal level of education did not correlate with vocabulary score in our sample, which was unexpected, given the frequent finding that children of mothers with higher levels of education have better language skills. We therefore decided to not use maternal level of education as the sole proxy for SES. Rather, we employed a composite SES measure, that included maternal and paternal levels of education, maternal and paternal employment status, household income, and expenditure on food. This composite allowed us to compensate for some missing data on sensitive questions^11^ regarding SES in the family background questionnaire. Each sensitive question in the family background questionnaire allowed for either “Don’t know” or “Don’t want to say,” which resulted in even answered questions sometimes rendering no data. The composite SES score was calculated out of 10: a SES composite score of 10, for instance, indicates that both parents studied beyond high school and are employed, whereas a score of 0 indicates that both parents are unemployed, that the household income is zero, and that the family has no money to spend on food, and are therefore reliant on food parcels provided by non-government or not-for-profit organizations. For all four languages combined, the SES range was 0 to 10, but as can be seen in Table 3, only the isiXhosa group displayed the full range. The Afrikaans group had a higher mean SES score than the isiXhosa and Xitsonga groups, and the SAE group had the highest score. This could be because many of the Afrikaans and SAE participants were recruited online and had access to electronic devices and good internet connections, which could be indicators of comparative affluence, and the SAE participants were almost exclusively from urban areas where remuneration is typically higher.

### RQ1 and RQ2: Sociocultural Factors as Predictors of Vocabulary Score

After ensuring that there was no multicollinearity between variables, the variables were regressed in separate models based on Bronfenbrenner’s (1977) ecological systems theory. Results of these models can be found in Table 4.

**TABLE 4:** Hierarchical multiple regressions of sociocultural predictors of total vocabulary score.

Block		*B*	SE	β	*R*^2^	Adjusted *R*^2^	Δ*R*^2^
1	Age	2.670	0.245	0.470***	0.221	0.219	0.221
2	Age	2.654	0.244	0.467***	0.231	0.227	0.010*
	Sex	−5.504	2.383	−0.099*			
3	Age	2.630	0.243	0.463***	0.243	0.232	0.012
	Sex	−5.681	2.390	−0.102*			
	Number of other children in household	0.829	0.887	0.042			
	Number of adults in household	1.363	0.903	0.067			
	Maternal education	2.955	1.709	0.102			
	SES	−0.390	0.854	−0.027			
4	Age	2.636	0.243	0.464***	0.252	0.238	0.010
	Sex	−5.491	2.389	−0.099*			
	Number of other children in household	1.042	0.919	0.053			
	Number of adults in household	1.152	0.905	0.057			
	Maternal education	2.973	1.777	0.102			
	SES	−1.226	1.040	−0.084			
	Language	0.177	1.263	0.007			
	Geographic area	6.630	2.895	0.116*			

***Significance at the 0.001 level.

*Significance at the 0.05 level. Maternal Education scale: 1 = no formal schooling, 2 = primary school incomplete, 3 = completed primary school, 4 = high school incomplete, 5 = completed high school, 6 = post-school qualification. SES composite score based on maternal and paternal level of education, maternal and paternal employment status, household income, and household expenditure on food.

The first model was statistically significant [*F*(1,420) = 119, *p* < 0.001], and accounted for 22.1% of the variance, with Age predicting Total vocabulary score with a high significance (β = 0.470, *p* < 0.001). The addition of Sex in Model 2 significantly predicted the Total vocabulary outcome (β = −0.099, *p* = 0.021), and this model significantly accounted for an additional 1% of the variance [*F*(2,419) = 599, *p* < 0.001]. The third block, containing the four microsystem factors, described a further 1.2% of the variance [*F*(6,415) = 595, *p* < 0.001]. The fourth block, which contained the two exosystem factors, explained an extra 1% of the variance [*F*(8,413) = 590, *p* < 0.001], where Geographic area significantly predicted Total vocabulary score (β = 0.116, *p* = 0.023), with rural-situated children having lower scores than urban-situated children. Although only Model 2 underwent a statistically significant improvement, the addition of each variable led to some improvement in the ability of the model to account for variation in vocabulary score. This is evidenced by the increase in adjusted *R*^2^ values with each new model (Model 1:0.219, Model 2:0.227; Model 3:0.232; Model 4:0.238), indicating that the improvement is not an artifact of the regression analysis but rather an effect of the addition of new variables improving the model fit. The final model as a whole accounted for 25% of the total variance in Total vocabulary score.

### RQ3: Relationships Between Sociocultural Factors and Semantic Domains

Due to the regression models (reported above) yielding a highly significant influence of age, age was partialed out of the correlations. Refer to Table 5 for the full output of the languages combined. Every factor was significantly correlated to one or more semantic domains. Notably, Language correlated with less than half of the domains, namely ANIMALS (*r* = −0.192, *p* < 0.001), VEHICLES (*r* = −0.098, *p* = 0.044), TOYS (*r* = −0.204, *p* < 0.001), and FOOD AND DRINK (*r* = 0.112, *p* = 0.022).

**TABLE 5:** Correlations of 10 semantic domains and sociocultural factors, of all languages combined.

	Animals	Vehicles	Toys	Food and drink	Clothes	Household items	Furniture	Places	People	Games and routines	Total
Sex	–0.085	0.035	–0.087	–0.052	−0.143**	−0.106*	−0.098*	–0.095	−0.128**	−0.098*	−0.112*
Area	0.194***	0.067	0.207***	0.048	0.107*	0.045	0.094	0.143**	0.025	0.083	0.114*
Language	−0.192***	−0.098*	−0.204***	0.112*	–0.026	0.054	–0.013	–0.037	0.034	0.075	0.027
Children (HH)	−0.124*	–0.072	–0.091	0.096*	0.032	0.099*	0.062	0.016	0.169***	0.067	0.058
Adults (HH)	–0.070	0.030	–0.046	0.130**	0.066	0.097*	0.089	0.031	0.130**	0.041	0.069
Maternal education	0.193***	0.084	0.165***	0.025	0.002	0.011	0.043	0.057	–0.042	0.098*	0.071
SES	0.245***	0.072	0.197***	–0.030	0.001	–0.067	0.030	0.090	–0.032	0.045	0.037

***Significance at the 0.001 level.

**Significance at the 0.01 level.

*Significance at the 0.05 level.

*Children (HH), number of other children in the household; Adults (HH), number of adults in the household.*

*Maternal Education scale: 1 = no formal schooling, 2 = primary school incomplete, 3 = completed primary school, 4 = high school incomplete, 5 = completed high school, 6 = post-school qualification. SES composite score based on maternal and paternal level of education, maternal and paternal employment status, household income, and household expenditure on food.*

To determine how languages differed from what was found in the combined correlations, correlations were performed for each language separately on those semantic domains that correlated with language (i.e., ANIMALS, VEHICLES, TOYS, and FOOD AND DRINK). Refer to Table 6 for the full output (with Total vocabulary score inserted for reference). The individual factor Sex significantly correlates with all semantic domains in Afrikaans, except for VEHICLES (*r* = −0.012, *p* = 0.899). Sex yielded no significant correlations in any other languages. The Exosystem factor Geographic area shows a correlation with some semantic domains for all languages, except for SAE. This was to be expected as the SAE group only contains three participants from rural areas. Interestingly, Geographic area only correlates with one semantic domain in Xitsonga (FOOD AND DRINK: *r* = 0.296, *p* = 0.004). The Number of other children in the household only shows a significant correlation in one domain, ANIMALS, in Afrikaans (*r* = −0.237, *p* = 0.013) and in isiXhosa (*r* = 0.212, *p* = 0.025). Number of adults in the household correlates with only one semantic domain for Afrikaans (ANIMALS: *r* = −0.212, *p* = 0.028), and one for isiXhosa (FOOD AND DRINK: *r* = 0.205, *p* = 0.031). Maternal level of education correlates with two semantic domains and only in the Afrikaans group (ANIMALS: *r* = 0.323, *p* < 0.001; TOYS: *r* = 0.218, *p* = 0.023). The final Microsystem factor, SES, is correlated with two domains in the Afrikaans group (ANIMALS: *r* = 0.365, *p* < 0.001; TOYS: *r* = 0.222, *p* = 0.021).

**TABLE 6:** Correlations of four semantic domains and sociocultural factors, per language.

Language	Sociocultural factors	Animals	Vehicles	Toys	Food and drink	Total
Afrikaans	Sex	−0.231*	–0.012	−0.235*	−0.226*	–0.270
	Geographic area	0.363***	0.143	0.343***	0.307**	0.368***
	Children (HH)	−0.237*	–0.168	–0.147	–0.154	−0.201*
	Adults (HH)	−0.212*	–0.004	–0.167	0.102	0.034
	Maternal education	0.323***	0.040	0.218*	0.063	0.123
	SES	0.365***	0.028	0.222*	0.047	0.137
English	Sex	–0.037	0.085	0.056	0.042	0.027
	Geographic area	–0.003	0.077	0.054	0.004	0.015
	Children (HH)	0.119	0.083	0.172	0.171	0.245*
	Adults (HH)	0.009	0.071	0.053	–0.004	0.058
	Maternal education	–0.021	–0.034	–0.047	–0.006	–0.033
	SES	–0.080	–0.117	–0.133	–0.126	–0.127
isiXhosa	Sex	–0.067	–0.064	–0.137	–0.073	–0.113
	Geographic area	−0.381***	−0.255**	−0.334***	−0.337***	−0.343***
	Children (HH)	0.212*	0.158	0.121	0.159	0.178
	Adults (HH)	0.004	–0.021	0.023	0.205*	0.091
	Maternal education	0.016	0.059	–0.033	0.027	0.092
	SES	–0.099	–0.077	–0.183	–0.025	–0.056
Xitsonga	Sex	0.014	0.129	–0.045	0.004	–0.125
	Geographic area	0.049	0.000	0.105	0.296**	0.313**
	Children (HH)	–0.194	–0.200	–0.084	0.070	0.022
	Adults (HH)	–0.065	–0.001	–0.059	0.095	0.007
	Maternal education	–0.075	–0.043	0.023	0.023	0.017
	SES	–0.102	–0.091	0.002	0.156	0.127

***Significance at the 0.001 level.

**Significance at the 0.01 level.

*Significance at the 0.05 level.

*Children (HH), bumber of other children in the household; Adults (HH), number of adults in the household.*

*Maternal Education scale: 1 = no formal schooling, 2 = primary school incomplete, 3 = completed primary school, 4 = high school incomplete, 5 = completed high school, 6 = post-school qualification. SES composite score based on maternal and paternal level of education, maternal and paternal employment status, household income, and household expenditure on food.*

## Discussion

### Effect of Sociocultural Factors on Vocabulary Size

As the first of its kind from South Africa, the current study set out to discover whether certain sociocultural factors relate to, and influence, vocabulary size and composition across four of South Africa’s official languages. Our first research question was whether there is an effect of sociocultural factors, divided into individual, microsystem and exosystem factors, on overall vocabulary size. Results showed that the child’s age was the strongest predictor, followed by the child’s sex, both of which are individual factors. The only other factor to significantly predict overall vocabulary size was geographic area.

The second research question asked whether combining all sociocultural factors in one model accounts for variance in the vocabulary outcomes. Findings indicate that the final model accounts for 25% of the overall variance, and that the addition of all sociocultural factors significantly improves the model. It should, however, be noted here that age (which is not a sociocultural factor) was the single variable which accounted for the most variance (22%). The CDI was developed as an assessment tool for measuring toddlers’ language development, so it stands to reason that age would be an important predictor of vocabulary size, as shown for a range of languages from different language families (Frank et al., 2021). However, at the microsystem level (considering the number of other children and adults in the household, maternal level of education, and SES), after controlling for the individual factors, a 1.2% increase in variance was found. The addition of the exosystem factors (home language and geographic area) accounted for another 1% of the variance. It can be concluded that the sociocultural factors we investigated – although shown in other published studies to affect language development significantly – are not particularly suited to predicting vocabulary size in our sample of children, and that age is the most important predictor. These findings will be discussed in more detail below.

### Effect of Sociocultural Factors on Semantic Domains

To answer the third research question, addressing vocabulary composition, 10 semantic domains of the CDI were selected for their assumed comparability across languages, that is to say, they are the most tangible items, or most common routines, to which the majority of children might be exposed. Findings were mixed for the different domains, with significant correlations for most factors apparent across at least one domain, though in the case of a factor such as sex or geographic area, a significant relationship was found across five and six domains, respectively (as shown in Table 6). This echoes the results of the findings from Research Question 1, which also showed sex and geographic area to be significant predictors of vocabulary size. Language was found to be significantly correlated with four semantic domains: ANIMALS, VEHICLES, TOYS, and FOOD AND DRINK. This required further investigation, and correlations were rerun separately in each language for those four semantic domains. Once the languages were scrutinized separately, it came to light that not many correlations remained within languages. For instance, there were no sociocultural factors significantly related to the SAE group for these four domains. This finding is not unexpected given that all but three respondents were from urban areas. However, in the isiXhosa group, all four domains were highly significantly correlated with geographic area (urban vs. rural), and ANIMALS was correlated with number of other children in the household, and FOOD AND DRINK with number of adults. Afrikaans patterned similarly to isiXhosa, although VEHICLES was not correlated with geographic area; ANIMALS was correlated with all sociocultural factors, and TOYS with every factor except number of other children and adults in the household. Xitsonga only showed significant correlations with geographic area in the FOOD AND DRINK domain; no other correlations with geographic area were found. Results could have patterned differently had other semantic domains (for instance ACTIONS (verbs), DESCRIPTIVE WORDS (adjectives and adverbs), TIME, or CONJUNCTIONS) been selected for inclusion.

The overarching aim of this study was to determine the effect of individual, microsystem and exosystem factors on the size and composition of children’s expressive vocabulary. Although findings are mixed overall, it can be said that sociocultural factors do play a part in vocabulary size but that, when looking closer at each semantic domain, more specific relationships emerge. A common finding across research questions is that geographic area is highly related to most semantic domains in the South African context. As one would expect, the child’s home language is not predictive of overall expressive vocabulary size, it is, however, related to vocabulary size in two semantic domains. The lack of a significant effect of home language highlights that although language and culture are related, they should not be conflated when examining variation in vocabulary size. The geographic area in which a child grows up (rural vs. urban) is more important to consider in this regard than a child’s home language. Finer analyses of the data might, however, reveal further differences between languages.

Our finding that geographic area is predictive of vocabulary size concurs with that of Bornstein and Cote (2005) and Vogt et al. (2015), namely, that there are differences in vocabulary size between rural and urban children. Bornstein and Cote found that children from rural areas in Argentina and the United States (but not in Italy) outperformed their urban peers. These differences may be related to the type of language rural vs. urban mothers use (see Camaioni et al., 1998 for Italian), with rural mothers using more directives and urban mothers more labels and descriptions. Whether such rural/urban differences in maternal speech exist in our contexts is still to be discovered. Vogt et al. (2015) found that location significantly predicted expressive vocabulary size. Children from urban areas had substantially larger expressive vocabularies than children from rural areas. They attributed this to SES as their urban sample had higher SES and higher levels of maternal education.

Our current results from four languages indicate that the languages pattern in different ways, and that sociocultural factors are related to different semantic domains in different languages. For example, the ANIMALS semantic domain seems to be the most sensitive to sociocultural factors. Looking at the correlations across languages, it is only sex and number of adults in the household that do not relate with ANIMALS. When each language was considered separately, ANIMALS in the Afrikaans group was related to every sociocultural factor, the only semantic domain patterning in this way. The isiXhosa group also shows relations between ANIMALS and geographic area and number of other children in the household. Although the Afrikaans and isiXhosa groups share geographic area as a common correlation, Afrikaans shows a highly significant positive correlation, whereas isiXhosa shows a highly significant negative correlation. In other words, there is a positive relationship between being from an urban area and good performance on the ANIMALS domain in Afrikaans, but the opposite pattern is found in isiXhosa.

A possible explanation for these findings may lie in the level of exposure children have to animals and representations of animals in different geographic environments below 30 months of age. In a study of 2- to 3-year-old isiZulu-speaking urban children, names of domestic animals such as *chicken* were produced by 78% of children, but names of wild animals like *lion* and *crocodile* were only produced by 8% and 3% of children, respectively (Kunene and Ahmed, 2016). Children who have early exposure to representations of animals in the form of toys or on television and in books (which is more likely in urban areas with more resources) will produce more animal names than children who have little exposure to animals (in under-resourced urban areas and rural areas) unless there is a greater variety of actual animals in rural areas. Therefore, crosslinguistic analyses should be undertaken with caution because controlling for sociocultural factors is imperative to prevent over- or underestimating a child’s performance, especially when compared to other languages which may have different patterns of influence from sociocultural factors. This can be seen especially when comparing the findings from the ANIMALS domain in SAE and Afrikaans, where SAE has no significant correlations with any of the sociocultural factors.

### Maternal Education, Family and Language Input

The children in this study were 16 to 32 months old. It could be that the correlations between sociocultural factors and vocabulary size change after toddlerhood, and that differences in maternal level of education or SES (for instance) come to account for more variation in vocabulary size later in the child’s life. This would require further investigation. In this study, maternal education did not explain differences in vocabulary size between 16 and 32 months. Previous studies show that children of mothers with higher levels of education demonstrate better language skills, including higher levels of expressive vocabulary, than their peers whose mothers have lower levels of education. There are several reasons for maternal level of education in our study not showing a correlation with vocabulary size: A higher level of maternal education itself does not cause larger child vocabularies; rather, maternal level of education affects the quality and quantity of language input the child receives. However, firstly, in many South African households in which mothers are present, they are not necessarily the primary caregivers of their biological children. Often a household does not have a nuclear structure but includes extended family. Grandmothers, aunts and older female children, both siblings and cousins, are often primary caregivers, and caregiving is culturally a joint responsibility usually among older female family members (McDaniel and Zulu, 1996; O’Laughlin, 1998). In some cases, children are raised by grandmothers, as mothers are migrant workers (see Hall and Posel, 2019). In cases where a female other than the mother provides the majority of the childcare, maternal level of education might be less relevant than primary caregiver level of education. A further reason for our finding of maternal level of education not influencing vocabulary size could be that language socialization practices (more so than maternal level of education) affect children’s vocabulary acquisition and that these practices are not directly related to maternal level of education in all South African contexts. This explanation can only be considered further once more information on language socialization practices in our context becomes available.

Havron et al. (2019) found that children with an older sister had better language skills than children with an older brother. These authors state that the finding of the presence of older siblings having a negative effect on a child’s language development (see, e.g., Peyre et al., 2016) may be due specifically to the presence of older brothers. In our study, we did not find a correlation between the number of other children in the household and vocabulary size. However, given Havron et al.’s (2019) finding, our correlations for number of other children in the household might present differently should the age and sex of these other children be taken into account. These analyses may reveal more complex relations between vocabulary size and the number, sex and possibly the age of the children who co-reside in one home.

There are not many research findings on the effect of the number of adults in the household on the language input that children receive (however, see Shneidman et al., 2013; Sperry et al., 2018). Soderstrom et al. (2018) compared the language experiences of toddlers who hear language from one or a small number of household members to those who hear language from multiple household members and found no difference in the language development of the two groups at 3 years old. The amount of language directed at the child at 2 years old also appeared not to differ between the two groups.

In line with Weisleder and Fernald (2013), Soderstrom et al. (2018) also found that children in households with many adults might overhear a lot of language (without having as much language directed at them), and only language directed at them predicts their language development. In contrast, Sperry et al.’s (2018) study showed that an increase in adults in the household contributed to an increase in the quantity of child-directed speech, across SES backgrounds, indicating that a more nuanced view of the relationship between SES and child-directed speech is required, one that considers not only differences across socioeconomic strata but also differences within various strata. Caselli et al. (1995) and Caselli et al. (1999) found that Italian-speaking children produce more social words than English-speaking children do and suggested that this difference reflects the tendency for Italians to live in close proximity to their extended family. Qualitative, instead of quantitative, analyses of the types of words children use in one/few-adult households as opposed to those in many-adult households may allow one to discover qualitative differences in the vocabulary composition of young South African children.

Other patterns of correlations between the sociocultural factors investigated in our study and vocabulary size could emerge if field studies examining the nature of language input and social interactions are undertaken. In this regard, it might be important to note that the majority of research findings on child-directed speech are based on Northern contexts. These research findings might not be applicable to South African contexts. Consider that only 46%, 39% and 38% of South African parents report naming objects, counting, and talking about a range of topics, respectively, when interacting with their children aged 3 years and younger, which could point to limited language input in terms of quality, regardless of the number of adults in the household providing the input. Nearly half (48%) of South African children have never read a book with a parent or guardian (Statistics South Africa, 2018). In fact, in 58% of South African households, there are no books, printed or otherwise (South African Book Development Council, 2016). In this regard, Bradley et al. (1988) found that the availability in the home of books and other resources that could potentially provide cognitive stimulation predicts better later social and cognitive outcomes (including better language skills). It could point to the need to investigate not only the number of adults in the household, but also the type of interaction that each has with the child and the language socialization practices present in the child’s household and community. These practices could vary from language community to language community, thereby in part accounting for the difference in the correlations between languages as regards semantic domains – a matter which requires investigation.

### SES and Vocabulary Size

It is not clear why in our study, unlike those conducted in many other contexts, no correlation was found between vocabulary size and SES. It could be that our composite SES score was not sensitive enough and should have included other measures, for instance of physical overcrowding of children’s dwellings. Children living in overcrowded conditions are likely to be exposed to disorganization in their environments (accompanied by high noise levels and other distractions), placing them at risk for developing a poor understanding and representation of temporal order (Flores, 2004). These conditions might also affect the quantity and quality of the language directed to the children, thereby affecting their vocabulary acquisition. Alternatively, our results might have differed had we not focused on structural aspects of the child’s home environment but on process-related aspects, such as parental responsiveness, stimulating behavior of adults during parent–child interaction, and the frequency of joint picture book reading, which Attig and Weinert (2020) found to be associated with SES across the first 2 years of the lives of the children in their sample: Mothers with lower SES interacted with their children less sensitively and in less stimulating manners than mothers with a higher SES. Moreover, parents with lower SES engaged in joint book reading less often with their child than parents with a higher SES. Studies systematically investigating the influence of SES on such processes which affect the child’s home learning environment have not yet been conducted in South African contexts.

However, one also needs to consider the possibility that SES and maternal education are not strongly correlated with input in all social contexts. In fact, Sperry et al. (2018)’s comparison of five US communities with different levels of SES showed that vocabulary input varied substantially within a single socioeconomic group and that a focus on maternal input without considering multiple caregivers and other bystander input underestimates language input in low-income environments. Furthermore, we would argue that rich oral cultures may provide extensive language input no matter the level of education of those who provide input to the child. At the same time, one should consider that more input does not necessarily result in a higher expressive vocabulary on the part of the child in cultures that discourage children from talking directly to, or in front of, adults. Heath (1983) makes these language socialization differences clear in her seminal study comparing working- and middle-class adult-child interactions where children in some communities may be observers to adult social interaction and talk rather than direct interlocutors from which speech is explicitly elicited – see also studies in Kenya (Blount, 1971); Papua New-Guinea (Ochs and Schieffelin, 1982), Canada (Crago et al., 1993), and Western Samoa (Ochs, 1984, 1988; Simonsen, 1990).

## Conclusion

Children develop language in a complex social world in which there is interaction between individual, microsystem and exosystem factors. This exploratory study attempted to identify which sociocultural factors affect child language development in young South African children growing up in a variety of contexts (rural and urban, varied SES, small and large households, well-educated and less well-educated mothers). We took a snapshot of children’s lives and of their vocabulary sizes, and also looked at the make-up of their vocabulary in terms of specific semantic domains. We found that the language that they speak affects their vocabulary to a lesser extent than the geographic area (rural vs. urban) in which they are raised. Given the biological and environmental risk factors that many South African children are exposed to concerning language development, it is important to determine which factors affect them most at which stage of their preschool lives, so as to allow for a more solid language base before they enter school. Language socialization and input in the home and community are especially important to investigate, given that schooling has been found to have a negligible effect on vocabulary size by the end of second grade (see Biemiller, 2006 for a discussion). Less than optimal language input at home can thus not necessarily be made up for in the school context.

Some of our results do not concur with those of existing studies, notable those on maternal level of education and SES. Maternal education and SES in our context may be less important than socialization practices and cultural conventions pertaining to child-directed speech. In Northern contexts, maternal level of education and SES often serve as proxies for the quality and quantity of language input that the child receives. However, in our context where categories of education are different and cultural norms could dictate when and how a child is spoken to and is expected to speak, maternal level of education and SES may not directly relate to the child’s language abilities as much as in other contexts. These findings raise the question of whether such broad measures are really useful in understanding factors that impact language development across cultures. Nevertheless, child language researchers in the South African context are operating in a comparative knowledge vacuum as regards child-directed speech, parental responsiveness to children’s vocalizations, and language socialization practices. We realize that without better knowledge of the processes shaping our children’s home learning environments, the study of sociocultural influences on child language development will remain challenging, because research findings generated in the North may have limited generalizability to South African contexts. This study attempted to generate context-specific findings, and the results indicate that a complex interplay of sociocultural influences on language development of young South African children may be present, and that these would require further consideration in a systematic manner if a fuller understanding of this interplay is to be gained.

## Data Availability Statement

The datasets presented in this article are not readily available because “In South Africa, one has to apply for permission to share data when one submits one’s application to the research ethics committee, and at that point one has to state who will be granted access to the data. Once ethical clearance has been granted, every request for access to the data requires a request from the PI to the REC for amendment of the ethics application. For this reason, data is not easily shareable.” Requests to access the datasets should be directed to FS, fs@sun.ac.za.

## Ethics Statement

The studies involving human participants were reviewed and approved by the Research Ethics Committee: Social Behavioural and Education Research, Stellenbosch University and the Faculty of Health Sciences Human Research Ethics Committee, University of Cape Town. Written informed consent to participate in this study was provided by the participants’ legal guardian/next of kin.

## Author Contributions

All authors apart from MM, NB, and SY, who joined the project later, contributed to the conceptualization of the study. FS, HB, HO, MW, MM, MN, MP, NB, OM, and SY were involved in data collection. MW performed the statistical analysis and together with FS wrote the first draft of the manuscript. Thereafter all authors revised the manuscript.

## Conflict of Interest

The authors declare that the research was conducted in the absence of any commercial or financial relationships that could be construed as a potential conflict of interest.

## Footnotes

1 In this paper, we use the full, prefixed names of indigenous languages of South Africa. Where the prefixed form appears as group name, it is to be read as referring to the speakers of that language - e.g., *isiXhosa group* refers to the isiXhosa-speaking group.

2 We acknowledge that the term ‘Bantu’ was misused during apartheid to refer to black South Africans. However, we use the scientific term ‘Bantu’ in this paper, albeit circumspectly, to refer to a large and significant language family on the African continent, with 11 languages of this language family being indigenous to South Africa.

3 The team received full Level I authorization from the MacArthur-Bates Board CDI Advisory Board for the development of CDI adaptations for all South Africa’s official languages.

4 As stated by Hoff (2003), SES accounted for 5% of the variation in vocabulary size between mid SES and high SES children, but when the effect of maternal speech was removed, SES accounted for only 1% of the variation.

5 Hearing more, and more complex, language leads to more advanced vocabulary and grammar in children (Huttenlocher et al., 2002). For instance, children who attend daycares with high levels of caregiver speech have been found to demonstrate better language development than their peers from daycares with high levels of peer speech (McCartney, 1984).

6 South Africa has a Gini coefficient of 0.625 and the second largest degree of inequality in distribution of family income in the world (The World Bank Group, 2021).

7 https://mb-cdi.stanford.edu/

8 For instance, for Afrikaans, there were two focus group discussions (one with professionals and the other with parents of young children), each in a different part of the country; and for isiXhosa, there were two focus groups with parents in rural areas, one focus group with early childhood development workers in an urban area, and individual interviews with eight parents of young children in the same urban area.

9 The two Bantu languages use a system of subject- and object-agreement (which references within the large noun-class system), and so pronouns are not used in the same way as in the two West Germanic languages. While pronouns do occur in both isiXhosa and Xitsonga, they have a different role and are used for, e.g., emphatic statements, and their construction is varied as they also use agreement. Pronouns therefore did not (in early pilot data) form a part of the vocabulary of children in our age range (see also Smouse, 2013 for isiXhosa).

10 As the Xitsonga data was collected in healthcare clinics in Limpopo Province, we also obtained permission from the Limpopo Department of Health.

11 Questions about level of education may for instance be sensitive in South Africa, where school careers are often ended prematurely for financial reasons (only 40% of the population completes high school; Statistics South Africa, 2012), and higher education is expensive and thus not within reach of everyone. Questions about employment status and earnings are likewise sensitive, because unemployment is rife at 43% (based on numbers provided by Statistics South Africa, 2020), and more employed persons with lower than higher levels of education experienced recent salary decreases (25% of those who did not complete high school vs. 10% of those with a degree).
